# Complex Laparoscopic Myomectomy with Severe Adhesions Performed with Proper Preventive Measures and Power Morcellation Provides a Safe Choice in Certain Infertility Cases

**DOI:** 10.1155/2016/4705790

**Published:** 2016-09-07

**Authors:** Jaime Alfaro-Alfaro, María de los Ángeles Flores-Manzur, Roberto Nevarez-Bernal, Rodrigo Ayala-Yáñez

**Affiliations:** ABC Medical Center, Mexico City, Mexico

## Abstract

Laparoscopic myomectomy offers a real benefit to infertile patients with uterine fibroids and peritoneal adhesions. The procedure requires a skilled surgeon and laparoscopy technique to minimize adhesion formation and other proven benefits. Restrictions arise since this procedure requires power morcellation for fibroid tissue extraction. Two years ago, the Food and Drug Administration in the United States of America (FDA) issued the alert on power morcellation for uterine leiomyomas, addressing the risk of malignant cell spreading within the abdominal cavity (actual risk assessment from 1 in 360 to 1 in 7400 cases). We review a 30-year-old female, without previous gestations, hypermenorrhea, intermenstrual bleeding, and chronic pelvic pain. Transvaginal ultrasound reports multiple fibroids in the right portion of a bicornuate uterus. Relevant history includes open myomectomy 6 years before and a complicated appendectomy, developing peritonitis within a year. Laparoscopy revealed multiple adhesions blocking uterine access, a bicornuate uterus, and myomas in the expected site. Myomectomy was performed utilizing power morcellation with good results. FDA recommendations have diminished this procedure's selection, converting many to open variants. This particular case was technically challenging, requiring morcellation, and safety device deployment was impossible, yet the infertility issue was properly addressed. Patient evaluation, safety measures, and laparoscopy benefits may outweigh the risks in particular cases as this one.

## 1. Introduction

Adhesions are abnormal peritoneal fibrotic bands with varying degrees of stiffness that connect two surfaces that are normally separated in the peritoneal cavity. They are responsible for locating the inflammatory reaction when there is an infection or foreign body carrying oxygen to ischemic tissue [1]. Many serious complications may be associated to adhesions such as chronic pelvic pain and small bowel obstruction and are associated to dyspareunia and secondary infertility [2]. Adhesion formation occurs in 90% of abdominal and pelvic surgeries, with a lower incidence when the surgery is performed laparoscopically.

After surgical removal of adhesions (adhesiolysis), they will form again in 85% of the cases. In a survey conducted among gynaecological surgeons in German hospitals, adhesions were believed to develop in 15% of cases after laparoscopy [3]; still, in symptomatic patients, removal of postsurgical adhesions requires a second surgical intervention with a high risk of new adhesion formation. In this situation, early precautions aimed at preventing postsurgical adhesions are of paramount importance [2]. Preventive measures that are currently being used include the adhesion barriers that help separate the peritoneum from damaged tissue for a minimum of 3–5 days [4].

Laparoscopic or open myomectomies are used for the removal of subserosal and intramural fibroids (types 4–7 in FIGO classification [5]). The advantages offered by the laparoscopic approach and use of these adhesions include shorter recovery time, decreased morbidity, and less adhesion formation [6]. Current medical reviews state that complex myomectomies include fibroids greater than 5 cm in diameter, the presence of 3 or more intraligamentary fibroids, and type 4 fibroids; these are associated with an increased surgical complication rate. Fibroids recurrence rate is similar in open or laparoscopic myomectomies [5, 7]. Myomectomy is an alternative to hysterectomy for women who wish to retain their uterus, or those that want to become pregnant. Removal of fibroids should be considered if they are thought to be associated with heavy menstrual bleeding, pelvic pain, and/or pressure symptoms [8]. The laparoscopic approach offers several benefits that include faster recovery, decreased blood loss, fewer adhesion formation, and significant cosmetic advantages. Laparoscopic myomectomy usually takes additional training, surgical suturing expertise, and specialized equipment; overall, this technique has advantages that clearly outweigh disadvantages [9]. Laparoscopic myomectomy has been reviewed and questioned since power morcellation is required for fibroid extraction through the small laparoscopic incisions. Ever since the FDA emitted serious recommendations, discouraging the use of power morcellation in laparoscopic procedures, due to the risk of malignant tumor spread [10], laparoscopic myomectomy requires various safety protocols and new devices, generating a preference for an open procedure, avoiding complex situations without this instrument, or employing new, cumbersome safety equipment. We still consider this surgery as highly useful as in this case.

## 2. Clinical Case

**Figure 1:**
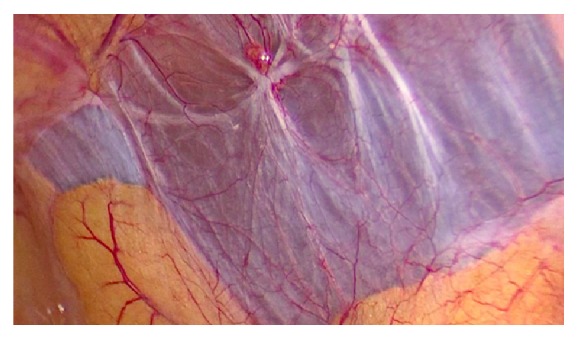
Right lateral pelvic wall adhesions, obstructing the proper identification of the uterus. Anatomic relations are of the upmost importance to proceed to adhesion removal.

**Figure 2:**
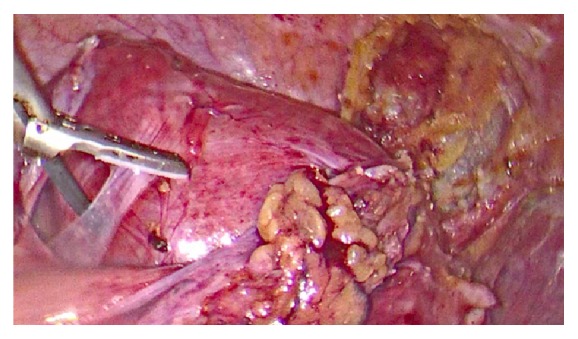
Bicornual uterus with dense adhesions to colon and various bowel segments. Proper resection of these adhesions had to be performed in order to have a clear view of the uterus and proceed to myomectomy.

We review a 30-year-old nulliparous female with hypermenorrhea, intermenstrual bleeding, and chronic pelvic pain for 4 months, prior to her office visit. A transvaginal ultrasound revealed a double uterus with a single cervix (bicornuate uterus) with the presence of 3 intramural leiomyomas (fibroids) in the right double uterus, measuring 41 × 33 × 33 mm, 57 mm, and 43 mm, respectively. The patient's relevant medical history included an open myomectomy 6 years ago with the removal of 4 leiomyomas, without the patient's knowledge of their location, and also a complicated appendectomy with peritonitis 1 year afterwards. She is programmed for laparoscopic myomectomy with previous bowel preparation and multidisciplinary evaluation in case of dense bowel adhesions. Bowel preparation was achieved with oral administration of sodium dibasic phosphate and 45 mL of sodium monobasic phosphate in half a glass of water, taking a separate, second glass of water 30 minutes later. Same dose was repeated 60 minutes later. Laparoscopy was performed placing an intraumbilical 5 mm trocar by direct vision and 1 accessory 5 mm trocar on the right and left lower pelvic quadrant, 3 cm above the iliac crest. Upon the introduction of the laparoscope multiple adhesions were observed (see Figure 1); the uterus was not visible upon initial evaluation. The left accessory trocar was placed and adhesiolysis was performed with ultrasonic energy instrument, in order to correctly identify and place the right accessory trocar. Once the adhesions were removed in the right lower quadrant of the pelvis, the accessory 5 mm trocar was placed as previously described. Removal of multiple adhesions from bowel to uterus had to be performed as well as from left and right ovaries and their respective Fallopian tubes (see Figure 2). Once the adhesions were removed, the bicornuate uterus was identified and leiomyomas were located on the right portion of the uterine body. We continued with adhesiolysis until the union of both uterine cavities was observed above the uterine isthmic portion. At this point, a diagnostic hysteroscopy was performed to identify both uterine cavities and exclude any types 1–3 leiomyomas. After the hysteroscopy, a conventional laparoscopic myomectomy was performed. Myomectomy was facilitated by the local infusion of argipressin (50 mg in 100 mL saline solution) around the fibroids.

**Figure 3:**
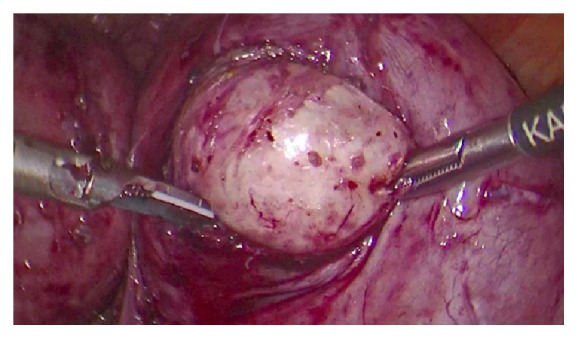
Leiomyoma enucleation in the fundal right segment of the bicornual uterus. Minimal invasive techniques were performed utilizing only two operative trocars with a third one at the umbilicus for the lens insertion.

**Figure 4:**
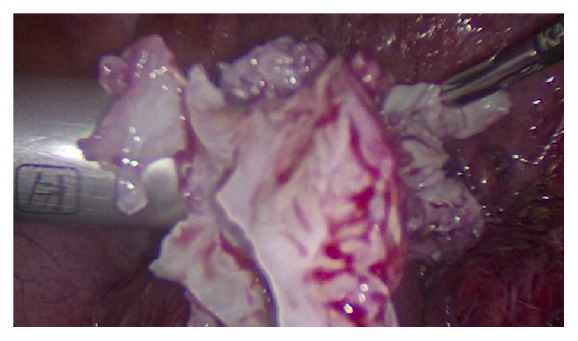
Power morcellation was employed to remove a total of 3 fibroids; the total morcellated leiomyoma mass was 101 g. Lack of such a crucial instrument would deprive patients like this one of benefiting from laparoscopy.

Incision of the pseudocapsule with ultrasonic scalpel was performed and traction with a toothed grasper assisted in the fibroid enucleation. The 3 fibroids were removed, and hemostasis was performed (see Figure 3). We proceeded to use the Rotocut 2 “Karl Storz” reusable power morcellator (see Figure 4) to extract the leiomyomas and the uterus was sutured in a three-layer approach.

Reoxygenated methylcellulose gelatin (Surgiflo®) and reoxygenated methylcellulose matrix (Surgicel Snow®) was placed on the uterine serosa with the intention of covering the barbed suture and preventing adhesion formation. The final pathology report identified the tissue as leiomyomas with a total morcellated weight specimen of 101 grams. Patient was discharged 20 h later without complications.

## 3. Discussion

Over half of the estimated 400,000 inpatient hysterectomies, performed every year in the United States, are done through laparoscopic techniques; thousands benefit from laparoscopic myomectomy; this particular procedure requires morcellation for the large mass extraction through a small orifice [11]. The case describes a complex myomectomy, further complicated due to a previous peritonitis, secondary to a complicated appendectomy. The bicornuate uterus does represent a further complication in the management of the laparoscopic myomectomy requiring a diagnostic hysteroscopy to identify and correctly place the uterine manipulator. Laparoscopy was opted due to the diminished adhesion formation, anatomic preservation, and improved fertility prognosis, and due to the patient's background we consider that the procedure outweighed the actual risks that morcellation may pose due to an unlikely myosarcoma.

Due to the 2014 FDA recommendations on power morcellation [10], this case could not have been performed through minimal invasive techniques in a hospital where power morcellation is banned. It is clear that the advantages offered by a laparoscopic myomectomy have been well documented with regard to patient's well being, length of hospital stay, and more relevant adhesion formation. Patient safety standards of care, regarding myomectomy, are preoperative preparation and appropriate presurgical evaluation when needed [11]. In complex cases, as this one, adequate preoperative preparations are in order, including bowel preparation and informed consent that conversion to open myomectomy is a possibility. Clinical cases such as this one benefit significantly from the laparoscopic myomectomy, indicating the use of power morcellation, with its respective cautions and proper preventive procedures. Currently, FDA is evaluating various containment devices for their use with power morcellation [12], allowing the marketing of containment systems to be used with certain morcellators and in selected cases [13].

It is important to remember that the FDA stated that the risk of spreading unsuspected uterine sarcoma is 1 in 350 to 1 in 7000 patients with the use of a power morcellation [2]. The following question is raised: has the FDA recommendation prevented patients from being offered a laparoscopic myomectomy in cases where it could have been of significant benefit? The concerns are real and enhanced malignancy detection should be implemented, although other morcellation techniques may be employed. In-bag power morcellation was a difficult option in this case since bag deployment was simply impossible due to the lack of space and adhesion process. We also must consider that the only randomized trial of in-bag manual versus uncontained power morcellation found no differences in total procedure time, morcellation time, simplicity, or operative complications in 104 women who underwent laparoscopic myomectomy [13, 14]. Have we been overly cautious or rightly so? Recognizing that the risk of dissemination is highly infrequent, laparoscopic myomectomy should not be discarded in this population, although a larger volume of cases will prove the risk and usefulness of both the myomectomy and the preventive procedures for malignant tissue spreading.

Complex adhesion cases pose a serious enough challenge to any laparoscopy expert, further complications arise without the use of power morcellation, and the cumbersome use of other devices may hinder the decision of performing this procedure laparoscopically, opting for an open variant and, hence, eliminating the benefits a minimal invasion procedure provides for infertility situations.

It is our conclusion that laparoscopic myomectomy, with power morcellation in cases such as these, with proper preventive controls, offers a real benefit for the infertile patient.
